# Emergence potential of mosquito-borne arboviruses from the Florida Everglades

**DOI:** 10.1371/journal.pone.0259419

**Published:** 2021-11-22

**Authors:** Durland Fish, Robert B. Tesh, Hilda Guzman, Amelia P. A. Travassos da Rosa, Victoria Balta, James Underwood, Charles Sither, Nikos Vasilakis

**Affiliations:** 1 Department of Epidemiology of Microbial Diseases, Yale School of Public Health, Yale School of the Environment, New Haven, Connecticut, United States of America; 2 Department of Pathology, Center for Tropical Diseases and Institute for Human Infection and Immunity, University of Texas Medical Branch, Galveston, Texas, United States of America; 3 Environmental Studies Program, Yale University, New Haven, Connecticut, United States of America; 4 Department of Entomology, North Carolina State University, Raleigh, North Carolina, United States of America; Fundacao Oswaldo Cruz Instituto Rene Rachou, BRAZIL

## Abstract

The Greater Everglades Region of South Florida is one of the largest natural wetlands and the only subtropical ecosystem found in the continental United States. Mosquitoes are seasonally abundant in the Everglades where several potentially pathogenic mosquito-borne arboviruses are maintained in natural transmission cycles involving vector-competent mosquitoes and reservoir-competent vertebrate hosts. The fragile nature of this ecosystem is vulnerable to many sources of environmental change, including a wetlands restoration project, climate change, invasive species and residential development. In this study, we obtained baseline data on the distribution and abundance of both mosquitos and arboviruses occurring in the southern Everglades region during the summer months of 2013, when water levels were high, and in 2014, when water levels were low. A total of 367,060 mosquitoes were collected with CO_2_-baited CDC light traps at 105 collection sites stratified among the major landscape features found in Everglades National Park, Big Cypress National Preserve, Fakahatchee State Park Preserve and Picayune State Forest, an area already undergoing restoration. A total of 2,010 pools of taxonomically identified mosquitoes were cultured for arbovirus isolation and identification. Seven vertebrate arboviruses were isolated: Everglades virus, Tensaw virus, Shark River virus, Gumbo Limbo virus, Mahogany Hammock virus, Keystone virus, and St. Louis encephalitis virus. Except for Tensaw virus, which was absent in 2013, the remaining viruses were found to be most prevalent in hardwood hammocks and in Fakahatchee, less prevalent in mangroves and pinelands, and absent in cypress and sawgrass. In contrast, in the summer of 2014 when water levels were lower, these arboviruses were far less prevalent and only found in hardwood hammocks, but Tensaw virus was present in cypress, sawgrass, pinelands, and a recently burned site. Major environmental changes are anticipated in the Everglades, many of which will result in increased water levels. How these might lead to the emergence of arboviruses potentially pathogenic to both humans and wildlife is discussed.

## Introduction

Florida has experienced a resurgence of mosquito-borne arbovirus activity in recent years primarily due to invasion by exotic viruses such as West Nile, dengue, chikungunya, and Zika [1–3]. However, there other are arboviruses that naturally occur in the Florida Everglades which have emergence potential due to impending environmental changes, such as wetlands restoration, climate change, introductions of exotic plants, mosquitoes and vertebrates. and suburban expansion. The southern Greater Everglades Region of South Florida is comprised of approximately one million hectares of nearly continuous wilderness located between the city of Naples on the west to the megalopolis of Miami and Ft Lauderdale to the east [4]. This expansive area is protected by several state and federal parks, preserves, and wildlife refuges and is inhabited by only a few small settlements of permanent residents, park staff, and scattered villages of Amerindians. However, more than 2 million tourists visit this area each year [5]. Despite being one of the world’s largest natural wetlands, little is known of the mosquito fauna or the diversity of arboviruses that occur in this region.

This is surprising since the region is undergoing a $10.5 billion wetlands restoration project designed to alter the hydrology and restore the flora and fauna to pre-disturbance levels, after decades of draining water for agriculture and residential development [6]. Restoration, as well as sea level rise, warming temperatures, invasive species and adjacent development are all anticipated to have a significant impact on the flora and fauna of the Everglades [7–9]. Accordingly, we conducted a systematic survey of the region to determine the diversity and abundance of the mosquito fauna and their associated arboviruses in order to provide baseline data and to determine the potential for arbovirus emergence that could affect human and animal health in South Florida as well as pose a threat to other regions of the US.

The earliest study of arboviruses in the Everglades was conducted by the Centers for Disease Control (CDC) in 1960 after an unusually high prevalence (80%) of antibody to Venezuelan equine encephalitis virus (VEEV) was observed in Seminole Indians living in the Everglades [10]. Further studies revealed a number of previously unknown arboviruses in the area also with significant antibody prevalence in the local Amerindian population [11–15].

A total of 10 arboviruses have been reported from the Everglades of which 5 are considered to be endemic (native) to this ecosystem. These include 4 species or subspecies in the genus *Orthobuyavirus*, Shark River virus (SRV), Mahogany Hammock virus (MHV), Gumbo Limbo viris (GLV) and Pahayokee virus (PAHV), and one *Alphavirus*, Everglades virus (EVEV). With the exception of EVEV, an endemic variant of VEE [16–18], very little is known about these viruses other than their original descriptions some 50 years ago. The other arboviruses reported from the Everglades have a much broader geographic distribution in the US and include West Nile virus (WNV) and St Louis encephalitis virus (SLEV) in the genus *Flavivirus*, Tensaw (TENV) and Keystone (KEYV) viruses in the genus *Orthobunyavirus*, and Eastern equine encephalitis virus (EEEV), an *Alphavirus*.

The landscape features of the Everglades vary, depending upon subtle changes in elevation which determine floristic assemblages adapted to flooding due to minor topographic relief. The highest elevation in Everglades National Park is less than 1 meter above sea level. Consequently, the landscape features are composed of strikingly discrete plant communities adapted to different elevations, ranging from sea level (mangrove) to persistently dry islands of limestone outcrops (hardwood hammock). Intermediate communities include cypress, sawgrass, and pineland, which are flooded in succession according to increasing elevation [19,20]. This gradient of uneven topographic relief results in a mosaic of discrete plant communities forming landscapes characteristic of the Everglades.

Arbovirus maintenance cycles in natural ecosystems are dependent upon the distribution and abundance of mosquito species capable of becoming infected and of transmitting virus among reservoir-competent hosts. The distribution and abundance of both mosquitoes and vertebrate hosts are dependent upon landscape features capable of supporting their populations. Mosquitoes invariably require an aquatic environment for larval development while most vertebrate hosts require terrestrial resources, both of which vary with water level. For example, flooded landscapes such as sawgrass are more favorable to wading avian hosts, while upland landscapes such as hardwood hammocks are more favorable for terrestrial mammals. Thus, arbovirus maintenance cycles are dependent upon specific landscapes that support their susceptible vector and host populations at different water levels.

The dynamic nature of the hydrology in the Everglades causes extreme fluctuations in the duration and frequency of ground water impoundments that determine larval habitat availability for different mosquito species, as well as the distribution and abundance of vertebrate hosts. The region experiences distinct wet (May-November) and dry (December- April) seasons causing up to 2 meters in annual fluctuation of the water table that sequentially inundate the landscapes each year according to their different elevations [19–21]. Interannual fluctuations in the water table may also vary several meters depending upon the level during previous years. Prolonged winter droughts and extreme summer/fall rainfall events, including hurricanes, are common and also affect the water table.

Here, we describe the distribution and prevalence of arboviruses in the Everglades and identify some mosquito species most likely responsible for viral maintenance during the summer seasons of 2013 and 2014. We also identify specific natural landscape settings where arbovirus activity is most prevalent in an attempt to elucidate the ecology of these little-known arboviruses and determine their distribution within the Greater Everglades ecosystem.

In order to assess potential emergence of these arboviruses, we have attempted to evaluate impending environmental changes in the region, such as hydrologic transformation resulting from wetland restoration efforts, climate change resulting in increased ambient temperatures and rising sea levels, the effect of invasive species, and the encroachment of residential development. Any or all of these environmental changes could alter the distribution and abundance of arboviruses increasing the exposure of both humans and wildlife to potentially pathogenic viruses.

## Methods

### Mosquito collection

**Fig 1:**
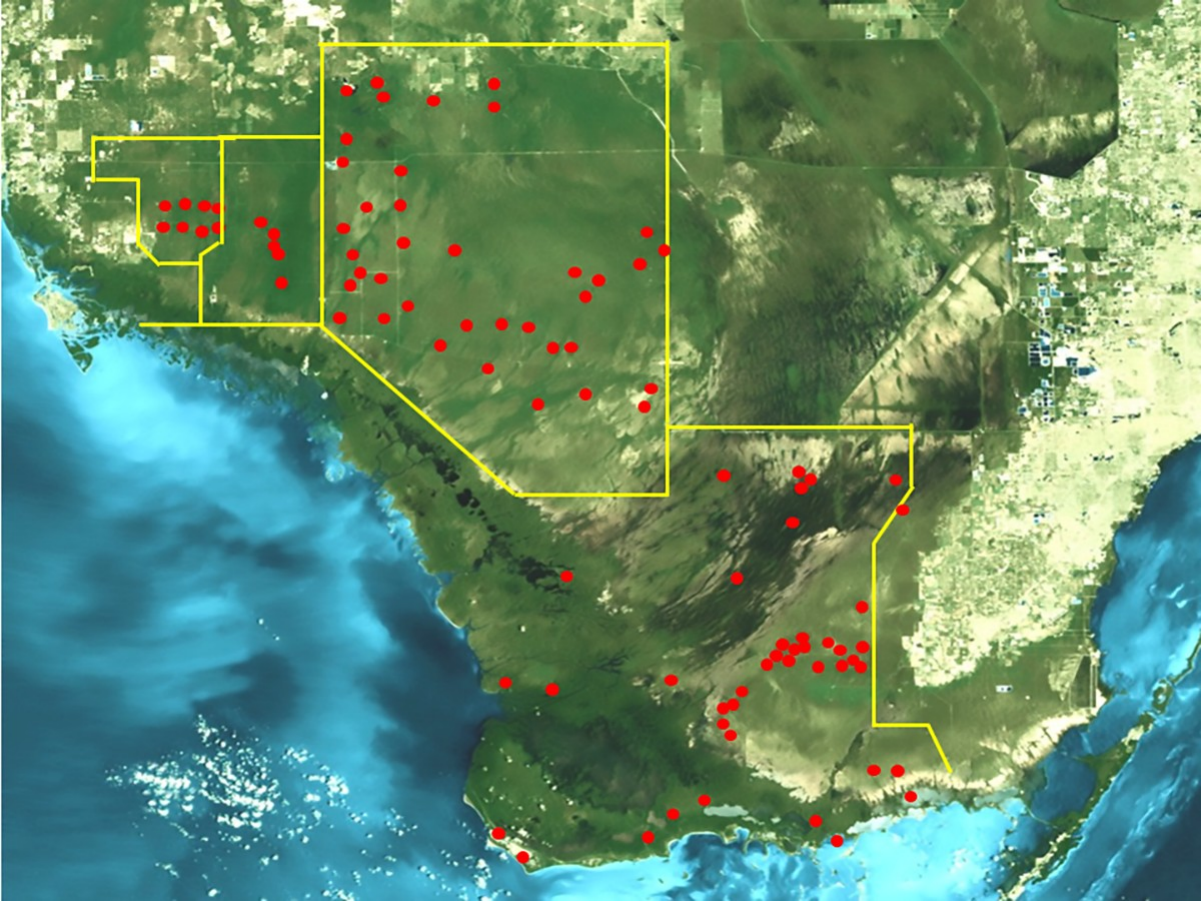
Collection sites. Composite Landsat 7 satellite image showing collection locations in Everglades National Park (EVER), Big Cypress National Preserve (BICY), Fakahatchee State Park Preserve, (FAK) Picayune State Forest Preserve (PY). Image courtesy of USGS/NASA Landsat.

**Fig 2:**
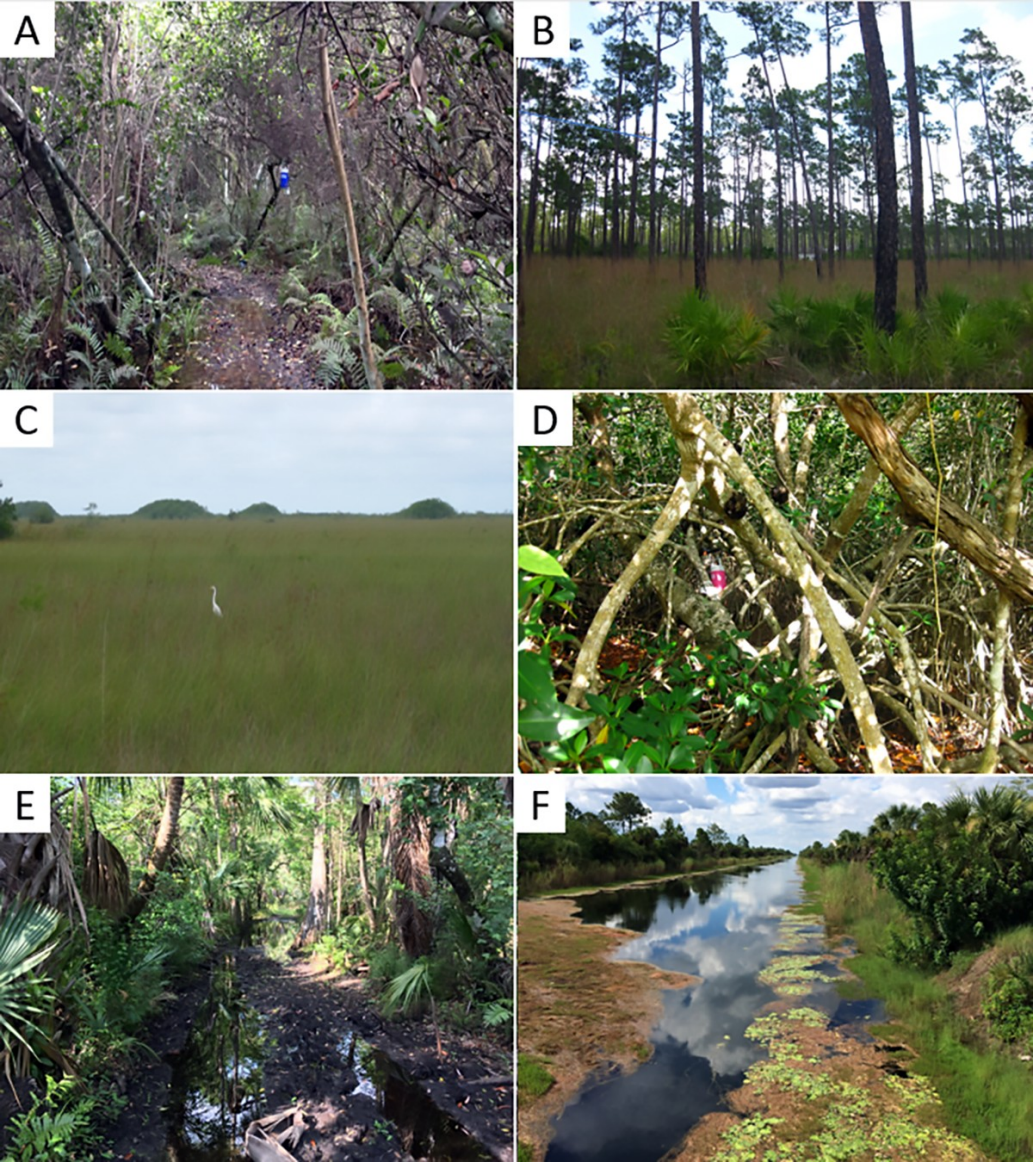
Landscapes sampled. (A) hardwood hammock (tree island), (B) pineland, (C) sawgrass with cypress domes in background, (D) mangrove, (E) Fakahatchee palm-cypress swamp, (F) Picayune restoration area.

A total of 105 study sites was sampled for mosquitoes during the summers (June-August) of 2013–14. The criteria for selecting sites were access and inclusion of dominant natural landscapes occurring within the southern Everglades region. Much of this region is inaccessible by means other than watercraft or specialized vehicles, so preference was given to sites with road or trail access. Attempts were made to sample spatially distant sites, but many were clustered due their accessibility. Priority was given to sampling different landscapes. The main landscape features in the southern Everglades can be classified into 5 dominant vegetation communities [19,22], which were sampled accordingly: Sawgrass (SG, N = 13), cypress (CY, N = 15), hardwood hammock (HH, N = 37), mangrove (MA, N = 15), and pineland (PL, N = 12), all of which occur in Everglades National Park (N = 54) and Big Cypress National Preserve (N = 38). Additionally, a palm-cypress swamp located in Fakahatchee State Park Preserve, [23] (FA, N = 5), and an adjacent mixed species restoration area [24], Picayune State Forest, (PY, N = 8), were also sampled (Figs 1 and 2 and S1 Table). A recently burned site (BN) in Big Cypress Preserve was sampled once.

Permits and approvals were obtained from the National Park Service (EVER-2013-SCI-0032, EVER-2014-SCI-0055, BICY-2015-SCI-0006) and Florida Park Service (07221414).

Mosquitoes were collected using CDC miniature light traps (Bioquip Products Inc., Rancho Dominquez, CA) baited with CO_2_. In 2013, CO_2_ gas was generated by placing approximately 0.68 kg of dry ice pellets in 2L insulated thermos containers affixed immediately adjacent to each light trap. In 2014, CO_2_ gas was generated from compressed gas cylinders with a regulator delivery setting of 500ml/min through a tube affixed directly to the trap. The calculated total CO2 gas delivered to each trap during the approximate 14 hr trapping period was nearly equivalent at 360L during 2013 and 352L during 2014.

Each site was sampled with one trap for just one night (18:00h-08:00h), although four sites were sampled in both years. Mosquito collections were cooled with ice packs and transported to a field laboratory for enumeration, identification, and pooling for subsequent virus isolation. Up to 1,000 mosquitoes from each trap were identified on a cold table (0°C) using published taxonomic keys [25,26] and separated into pools of up to 50 mosquitoes from each site. Mosquito pools were stored (-80°C, <30d) until shipped overnight on dry ice to the World Reference Center for Arboviruses at University of Texas Medical Branch, Galveston TX. Voucher specimens of each mosquito species collected have been deposited at the South Florida Specimen Management Center at Everglades National Park (S2 Table).

### Virus isolations and identifications

Mosquito pools were thawed and homogenized in 2.0 mL of phosphate-buffered saline (pH 7.4) with 25% fetal bovine serum, using a TissueLyser (Qiagen, Valencia, CA) and 3 mm stainless steel beads. After centrifugation at 10,000 rpm in a micro centrifuge for 10 minutes, 150 uL of the supernatant was inoculated into 12.5 cm flasks with monolayer cultures of Vero E6 (ATCC CRL-1586) and C6/36 (ATCC CRL-1660) cells originally obtained from the American Type Culture Collection (ATCC). Cultures were maintained at 37°C and 28°C, respectively, for 10–12 days and were examined every 2 days for evidence of viral cytopathic effect.

Cultures showing viral cytopathic effects in Vero cells were harvested; and the culture fluid was tested by hemagglutination inhibition and complement-fixation tests against a battery of genius and serogroup-specific hyperimmune mouse ascitic fluids, obtained from the World Reference Center for Emerging Viruses and Arboviruses. The serological methods and preparation of hyperimmune mouse ascitic fluids are classical procedures and have been described previously [27,28]. RNA from some of the bunyavirus samples was extracted, reverse transcribed and sequenced on an Illumina HiSeq2000 as described previously [29] for confirmation. All isolates of vertebrate viruses cultured in Vero cells have been deposited in the National Park Service Special Collection at the World Reference Center for Emerging Viruses and Arboviruses, University of Texas Medical Branch (S3 Table).

### Data analysis

Entomological data were pooled for all mosquito species collected each year to calculate overall mosquito species composition and their relative abundances among landscapes. Relative mosquito abundance was calculated as a percentage of the total collected each year and for each landscape sampled. One-way ANOVA was conducted to determine significant differences among mosquito species within landscape types in Prism [30].

Arbovirus composition and prevalence were similarly calculated for each year and landscape. Maximum likelihood estimates (MLE) were used to calculate minimum infection rates (MIR) per 1,000 female mosquitoes from the mosquito pool data [31]. Data were analyzed using an add-in for Microsoft Excel available from CDC [32].

Hydrologic data on water levels were obtained from the South Florida Water Management District DBHYDRO website [33] Data from station site Loop1_T was selected because of its central location near the border of Everglades National Park and Big Cypress Preserve. Downloaded data consisted of average daily water level measured in feet (NGVD29) for the months May 1–Aug 31 for 2013 and 2014 and converted to centimeters.

## Results and discussion

**Fig 3:**
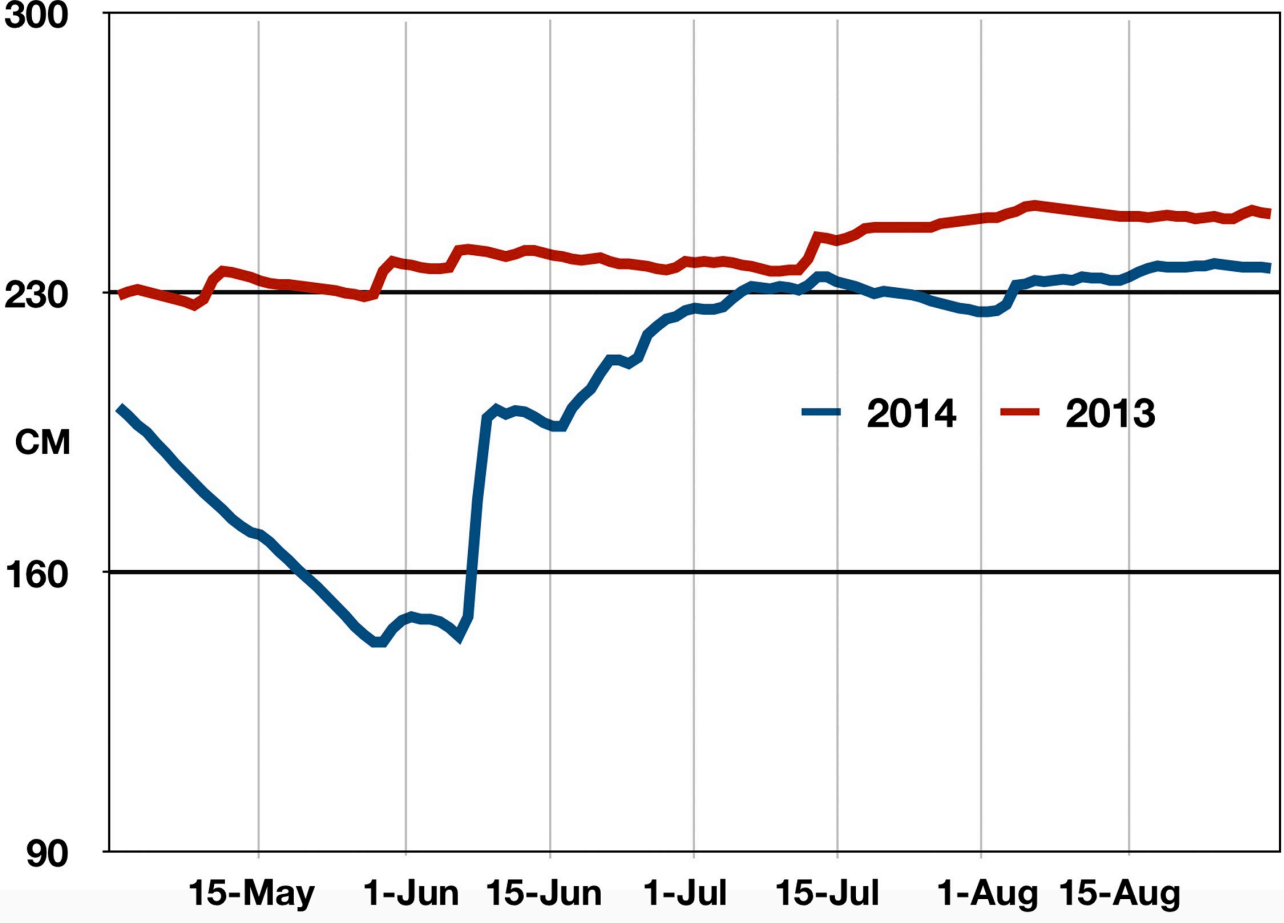
Water level. Summer water levels at Loop Road hydrologic station in 2013 and 2014 [33].

During the summer of 2013, higher than normal rainfall caused water levels to average 34.0 cm (1.1 ft) higher than in 2014 [33] (Fig 3). Maximum differences occurred at the beginning of the rainy season during late May and early June with a maximum difference of 95 cm (3 ft) on June 6. This difference in water level resulted in greater and earlier flooding of landscapes in 2013 compared to 2014, causing major changes in mosquito species composition and arbovirus activity between these two years. A comparison between years of markedly different water levels provide valuable insight into the dynamic nature of mosquito and arbovirus activity in the Everglades and illustrates the impact of water level change upon arboviral emergence.

### Mosquito diversity and abundance

**Fig 4:**
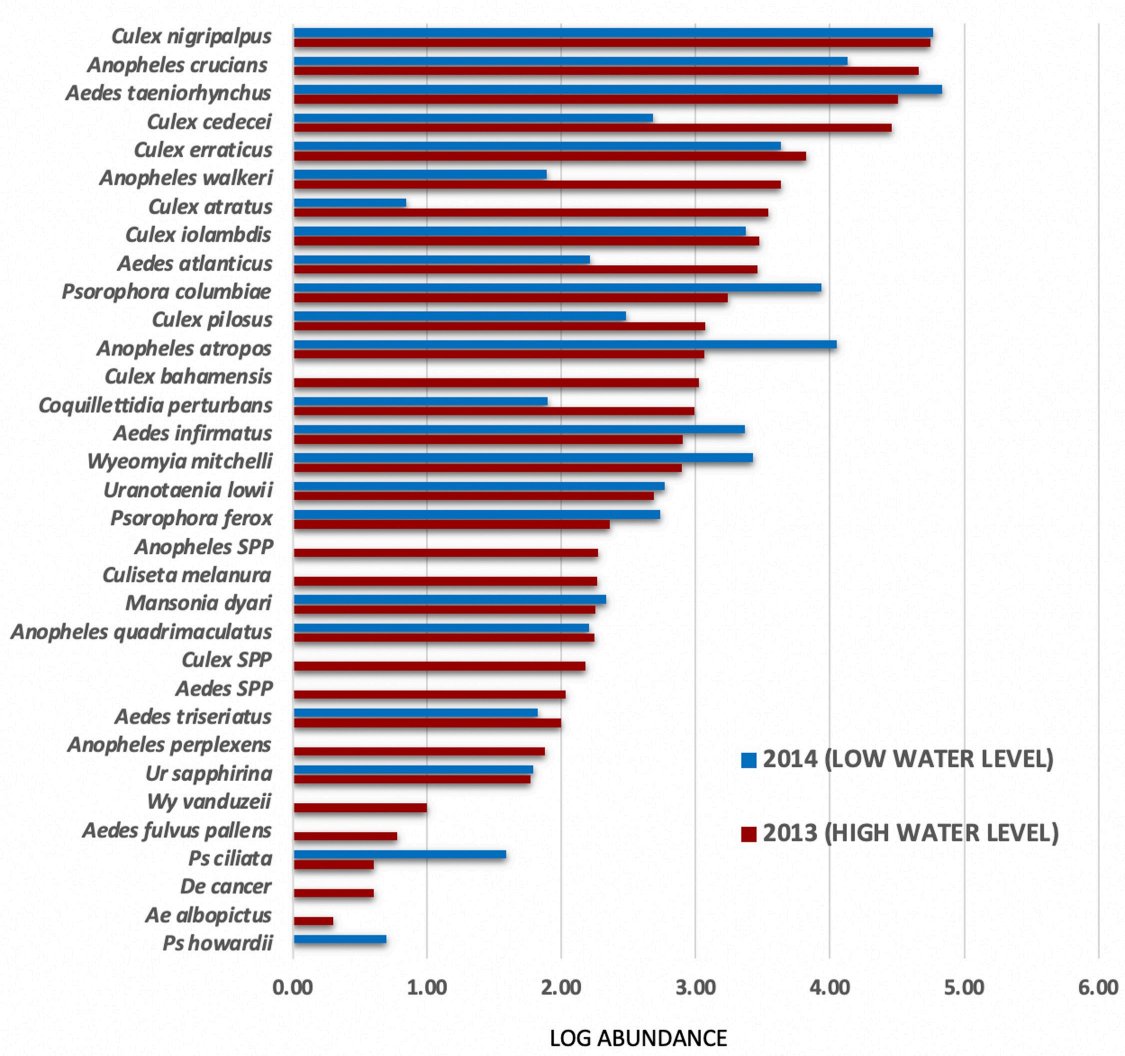
Mosquito abundance. Log abundance of each mosquito species collected in 2013 and 2014.

A total of 367,060 mosquitoes was collected during this study (192,440 in 2013, 174,620 in 2014) from 105 study sites (59 in 2013, 46 in 2014) (S4). The mean number of mosquitoes collected per trap night was 3,336 (3,261 in 2013 and 3,344 in 2014). Although 30 mosquito species were identified, only 10 species comprised 90% of the total collection and just 3 species (*Aedes taeniorhynchus*, *Culex nigripalpus* and *Anopheles crucian*s, a species complex [34]), comprised 75% of the total mosquitoes collected. The abundance and ubiquitous nature of the dominant mosquito species mask the abundance trends and distribution patterns of the less common species, as *Culex cedecei*, an important vector of some arboviruses (Fig 4).

**Fig 5:**
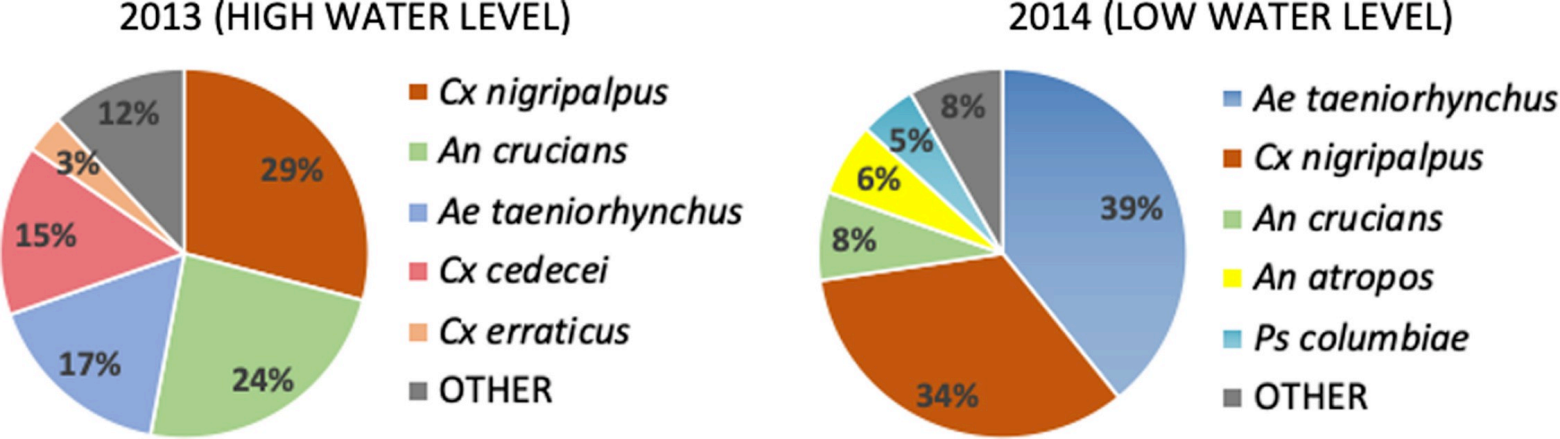
Mosquito species composition. Total mosquito species composition during 2013 (N = 192,440) and 2014 (N = 174,620).

Mosquito species composition and abundance varied considerably between the 2 years. Species dominance was similar in both years with the same 3 species (*Ae*. *taeniorhynchus*, *Cx*. *nigripalpus* and *An*. *crucians*) being most abundant (Fig 5).

During 2014, the abundance of *Ae*. *taeniorhynchus* more than doubled from 2013 while *An*. *crucians* decreased 3-fold and *An*. *atropos* increased 10-fold. *Culex cedecei* was relatively abundant in 2013 (15% of total) but rare in 2014 (<2%); likewise the abundance of *Cx*. *erraticus* decreased by 35%. *Culex nigripalpus* abundance was similar in both years. There were fewer species collected in 2014 (23) compared to 2013 (29), despite the nearly equal number of mosquitoes collected (174,620 vs 192,440 respectively) and number of colleting sites (46 vs 59).

Nine of the 10 dominant species are either proven or suspected vectors of arboviruses. *Aedes aegypti* was not collected during this study, as none of the study sites were located near human habitation where hosts and artificial containers for larval development would occur. *Aedes albopictus* was found in low numbers at a single camping site located in Everglades National Park. Although principal vectors of human pathogens, neither of these species are important vectors of zoonotic pathogens.

Mosquitoes are a major feature of the Florida Everglades for both residents and visitors. With a mean abundance of 3,336 mosquitoes/trap night, the abundance is >10X more than that reported for mosquito collections in the State of Connecticut [35], which employs a similar mosquito and arbovirus surveillance program with 89 site locations. Considering a conservative estimate of a 0.4 hectare catchment area for each light trap [36], the total number of mosquitoes in the southern Greater Everglades Region study area was estimated to be 7.3 billion in mid-July. Exposure to mosquito bites for humans and wildlife during the summer months is intense and unavoidable.

**Fig 6:**
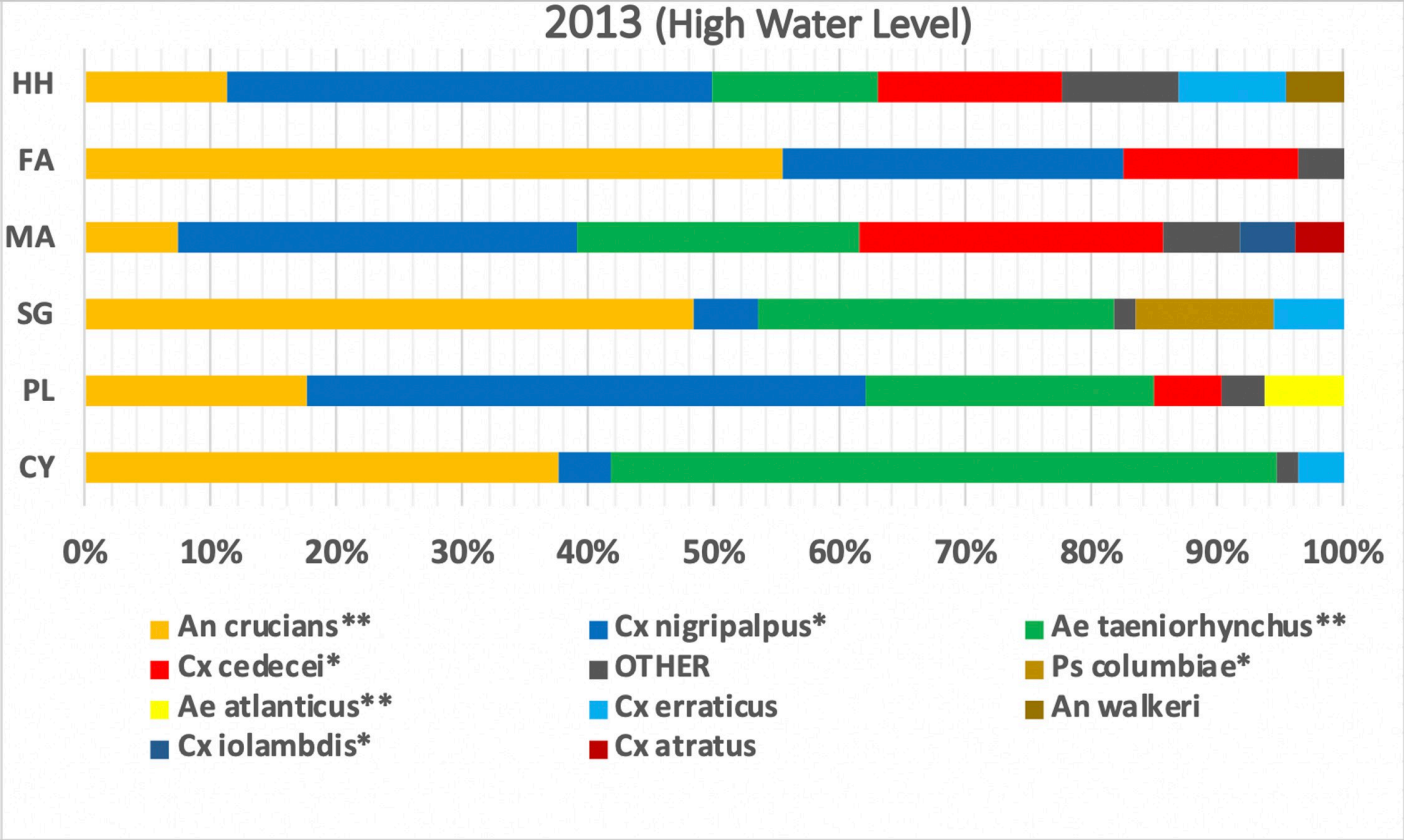
Distribution of mosquito species among landscapes during 2013. Hardwood hammock (HH), Fakahatchee (FA), mangrove (MA), sawgrass (SG), pinelands (PL), cypress (CY). One-way ANOVA levels of significant difference: *P<0.05, **P<0.01.

Mosquito species composition varied considerably among landscapes. The ubiquitous species, *Ae*. *taeniorhynchus*, Cx. *nigripalpus*, and *An*. *crucians*, were present in all landscapes during 2013, except Fakahatchee (Fig 6). In contrast, *Cx*. *cedecei* was most abundant in hardwood hammocks, Fakahatchee, and mangrove, but absent from sawgrass and cypress. *Culex erraticus* was most abundant in hardwoods hammocks and *Psorophora columbiae* was most abundant in sawgrass.

**Fig 7:**
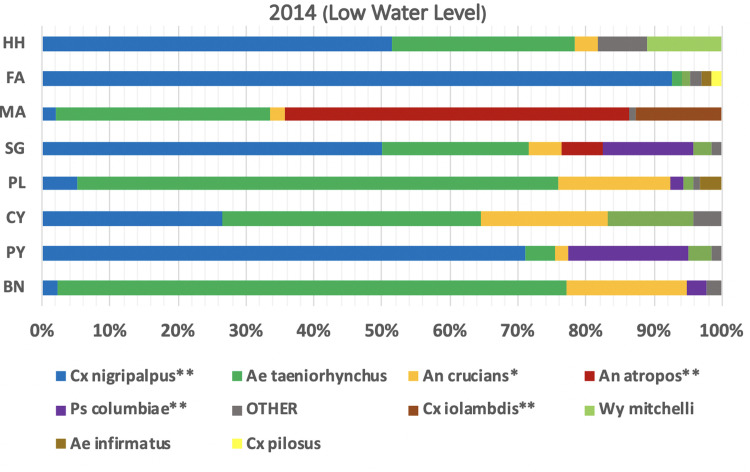
Distribution of mosquito species among landscapes in 2014. Hardwood hammock (HH), Fakahatchee (FA), mangrove (MA), sawgrass (SG), pineland (PL), cypress (CY), Picayune (PY), and burned site (BN). One-way ANOVA levels of significant difference: *P<0.05, **P<0.01.

During 2014, *Cx*. *nigripalpus* dominated nearly all of the landscapes and comprised more than 90% of the total in Fakahatchee compared with only 28% in 2013 (Fig 7). *Cx*. *cedecei* was rare (<1%) in all landscapes during 2014. *Culex iolambdis*, rare in 2013, was common in mangroves during 2014. *Anopheles atropos* was rare in all landscapes in 2013, but was the dominant species in mangrove during 2014 (Fig 7). Changes in the distribution of mosquito species during high water and low water determine the abundance of species capable of transmitting arboviruses among landscapes.

### Arbovirus diversity and abundance

**Fig 8:**
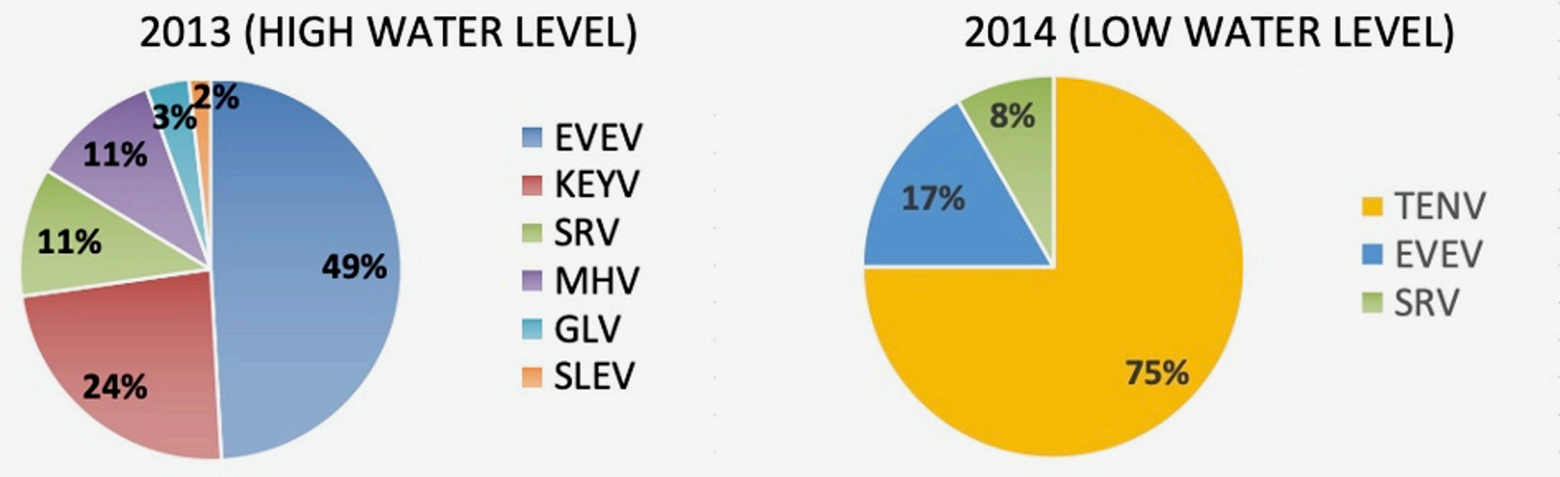
Arbovirus isolations. Composition of all vertebrate arboviruses isolated from mosquitoes in 2013 (N = 56/1037 pools) and 2014 (N = 12/973 pools).

In total, 70,941 mosquitoes were separated into 2,010 pools that were cultured for presence of arboviruses. Seven different vertebrate arboviruses were isolated in Vero cells from 68 mosquito pools. (Fig 8). None of these pools yielded mixed infections. Five of the isolated arboviruses (*i*.*e*. Everglades EVEV, Gumbo Limbo GLV, Mahogany Hammock MHV, and Shark River (SRV) are considered to be endemic to the Florida Everglades. Keystone virus (KEY), Tensaw virus (TEN) and St. Louis encephalitis virus (SLEV) are more widely distributed in North America. [37–39]. In addition, 161 isolates of presumed insect-specific arboviruses were isolated from C6/36 cells. Of these, three novel insect-specific viruses (Coot Bay virus, Big Cypress virus and Long Pine Key virus) were identified and described elsewhere [29,40,41].

The distribution of arboviruses was found to be highly variable, both among mosquito species and landscapes (Tables 1–3). *Culex cedecei* ranked fourth in abundance among the 30 species found (Fig 4) but ranked first in the number of virus isolations (Table 1).

**Table 1:** Arboviruses isolated from all mosquito species, 2013–2014.

SPECIES	EVEV	KEYV	TENV	SRV	MHV	GLV	SLEV	TOTAL
*Cx*. *cedecei*	16	1	3	6	6	2		34
*Ae*. *atlanticus*	1	9						10
*Cx*. *nigripalpus*	7	1					1	9
*An*. *crucians*			6					6
*Cx*. *atratus*	2			1				3
*An*. *atropos*			2					2
*Ae*. *taeniorhynchus*			1					1
*Cx*. *quinquefasciatus*	1		0					1
*Wy*. *mitchelli*	1							1
*Cx*. *erraticus*		1						1
TOTAL	28	12	12	7	6	2	1	68

**Table 2:** Minimum infection rate per 1,000 mosquitoes (95% CI) among landscapes 2013.

LANDSCAPE	EVEV	KEYV	SRV	GLV	MHV	SLEV
HH	1.04 (0.63–1.60)	0.51 (0.25–0.94)	0.23 (0.07–0.74)	0.11 (0.02–0.37)	0.23 (0.07–0.54)	0.06 (0.00–0.27)
FA	1.22 (0.04–2.94)	0.00 (0.00–1.12)	0.00 (0.00–1.12)	0.30 (0.02–1.46)	0.30 (0.02–1.46)	0.00
MA	0.18 (0.03–0.59)	0.09 (0.01–0.44)	0.27(0.07–0.74)	0.00	0.09 (0.01–0.44)	0.00
PL	0.74 (0.20–2.01)	0.59 (0.09–1.62)	0.00	0.00	0.00	0.00
CY	0.00	0.00	0.00	0.00	0.00	0.00
SG	0.00	0.00	0.00	0.00	0.00	0.00

HH = hardwood hammock, FA = Fakahatchee, MA = mangrove, PL = pineland, CY = cypress, SG = sawgrass. N = 192,440 mosquitoes, 1037 pools, 56 isolates.

**Table 3:** Minimum infection rate per 1,000 mosquitoes (95% CI) among landscapes 2014.

LANDSCAPE	EVEV	TENV	KEYV	SRV	GLV	MHV	SLEV
HH	0.54 (0.03–2.63)	0.00	0.00	0.19 (0.03–0.62)	0.00	0.00	0.00
FA	0.00	0.00	0.00	0.00	0.00	0.00	0.00
MA	0.00	0.00	0.00	0.00	0.00	0.00	0.00
PL	0.00	0.20 (0.01–0.96)	0.00	0.00	0.00	0.00	0.00
CY	0.00	0.62 (0.16–1.67)	0.00	0.00	0.00	0.00	0.00
SG	0.00	1.25 (0.22–4.12)	0.00	0.00	0.00	0.00	0.00
BN	0.00	4.67 (0.85–15.79)	0.00	0.00	0.00	0.00	0.00
PY	0.00	0.00	0.00	0.00	0.00	0.00	0.00

HH = hardwood hammock, FA = Fakahatchee, MA = mangrove, PL = pineland, CY = cypress, SG = sawgrass, BN = burned, PY = Picayune. N = 174,620 mosquitoes, 973 pools, 12 isolates.

*Culex cedecei* is in the *Melanoconion* subgenus of *Culex*, a primarily tropical subgenus that also includes *Cx*. *atratus*, *Cx*. *iolambdus* and *Cx*. *erraticus*, species also found in this study [42]. Members of this subgenus are important vectors of arboviruses throughout the Neotropics, especially members of the VEEV complex [43]. *Culex cedecei* pools yielded 34 isolations of four arboviruses (EVEV, GLV, MHV, SRV) from 14 locations, primarily hardwood hammocks and Fakahatchee (Table 1). The overall infection prevalence in *Cx*. *cedecei* was 4.54/1,000 (CI = 3.15–6.36) in 2013, and 13.51/1,000 (CI = 3.5–38.1) in 2014. This single species appears to play a key role in maintaining several arboviruses, but little is known of its ecology in the Everglades.

Among the landscapes sampled during 2013, arbovirus prevalence was greatest in hardwood hammock, Fakahatchee, mangrove, and pineland respectively, and absent in cypress and sawgrass (Table 2). Hardwood hammocks also yielded the greatest diversity of arboviruses (6). Excluding the widespread activity of Tensaw virus during 2014, arboviruses (2) were found only in hardwood hammocks in 2014 (Table 3). Interestingly, TENV was not found in hardwood hammocks, mangrove, or Fakahatchee in 2014, but was present in cypress, sawgrass, pinelands, in addition to the burned site. More studies are needed to determine the pattern of landscape relationships among these arboviruses, but it is clear from this study that hardwood hammocks and Fakahatchee are important landscapes for the presence of most arboviruses, while cypress and sawgrass are not. However, the reverse seems to be true for TENV. No arboviruses were found at the Picayune restoration site (Table 3).

Despite their abundance (75%), *Cx*. *nigripalpus*, *Ae*. *taeniorhynchus*, and *An*. *crucians* yielded only 16 (24%) of the 68 total arbovirus isolates while 4 less common species (11.3%) *Cx*. *cedecei*, *Cx*. *attratus*, *An*. *atropos*, and *Aedes atlanticus* yielded 72% (Table 1). Minimum infection rates ranged from a low of 0.04/1000 (CI = 0.00–0.17) for SLEV in *Cx*. *nigripalpus* to 5.59/1000 (CI = 2.75–9.26) for KEYV in *Ae*. *atlanticus*. The overall minimum infection rate for all mosquito species in 2013 when water level was high was 1.43/1000 (CI = 1.09–1.84), but only 0.36/1000 (CI = 0.20–0.61) in 2014 when water level was low. The overall risk of infection from mosquito bites in the Everglades is highly species dependent. Species-specific infection rates are provided in S5 Table.

Several sites were hot spots of virus activity. A single light trap collection from a hardwood hammock in Everglades National Park yielded 6 arbovirus isolates (3 KEYV, 1 EVEV, 1 GLV, 1 MHV), (MIR = 12.19/1000, CI = 5.77–29.73) on July 13, 2013, and a seventh (SRV) from a subsequent collection on Aug 7, 2013 (MIR = 1.28/1000, CI = 0.07–6.24). A second nearby hardwood hammock site yielded 5 isolates (4 EVEV, 1 KEYV) on June 20, 2013 (MIR = 9.37/1000, CI = 0.3.54–21.06) and 5 additional isolates (3 KEYV, 1 EVEV, 1 GLV) one month later on 24 July (MIR = 4.36/1000, CI = 1.43–10.60). Single isolates of EVEV and SRV were obtained from this site the following year on August 6, 2014 (MIR = 2.77/1000, CI = 0.16–13.58). Similarly, of 5 light trap collections obtained from the Fakahatchee on Aug 10, 2013, 4 yielded 6 isolates (EVEV (4), GLV (1), SRV (1) (MIR = 1.98/1000, CI = 0.77–3.87). All of these hot spots of virus activity were located along tourist trails readily accessible to the public.

A hot spot of virus activity for Tensaw virus was observed at the burned site during 2014. Wildfires caused by lightning strikes are a common occurrence in the Everglades [44]. *Anopheles crucians*, was second in abundance at this site (Fig 7) and is a known vector of TENV [45]. Having recently been burned, most of the original emergent vegetation was absent so the trap likely attracted *An*. *crucians* from a greater distance than normal from the surrounding sawgrass landscape. The high prevalence of TENV at this site and its broad distribution among other landscapes during 2014 (Table 3) suggests that transmission of this arbovirus is episodic as it was not found at any site during 2013.

Everglades virus (EVEV) was the most common arbovirus found in this study with 28 isolates from 5 mosquito species, mostly *Cx*. *cedecei*, from 14 locations (Table 3). Seven isolates were also obtained from *Cx*. *nigripalpus* the most abundant mosquito found in this study. EVEV is a member of the genus *Alphavirus*. Taxonomically, it is a member of the Venezuelan equine encephalitis complex of arboviruses, members of which have previously caused epidemics in humans and horses in South and Central America, and in Texas [46]. Rodents, especially the cotton rat (*Sigmadon hispidus*), are thought to be the primary reservoir hosts of EVEV [18,47]. Epizootics of EVEV have occurred in northern Florida, far outside of the range of *Cx*. *cedecei*, as is evidenced by positive canine serology [17]. Five cases of clinical illness attributed to EVEV infection have been described in South Florida [48,49] and high levels of EVEV antibodies (>80%) were observed in Seminole Indians living in the Everglades Work (10). Clinical manifestations of EVEV infections include high fever, severe headache, prostration, myalgia, and central nervous system signs, including encephalitis [50].

Tensaw virus is a species in the genus *Orthobunyavirus* and is widely distributed in the southeastern US [51]. It has been most frequently isolated from *Anopheles spp*. and small and medium sized mammals. Infection with TENV is not known to cause disease in humans, but a serological survey of 300 south Florida residents revealed that 22% had experienced apparent asymptomatic infection [52]. A recent epidemiologic study of congenital effects of arbovirus infections in pregnant women in Florida has suggested a correlation between TENV antibody and birth defects (microcephaly) [53].

Keystone virus (KEYV) is another species in the genus *Orthobunyavirus* [51]. KEYV was first isolated in 1964 from *Aedes atlanticus* in Tampa, FL [54]. The virus is widely distributed in the southeastern US, and *Ae*. *atlanticus* is the only known vector [39,55,56]. Serological studies of Tampa Bay area residents in 1972 revealed a 19 to 21% antibody prevalence in the human population, suggesting a high incidence of asymptomatic infection [39]. The first known clinical case of KEYV infection in a human occurred in 2016, when the virus was isolated from a Florida patient thought to have Zika virus infection [57]. Clinical symptoms were mild and included fever and rash [50].

Shark River virus (SRV) is a subspecies of Patois virus (PATV) and a member of the genus *Orthobunyavirus* [51]. It was first isolated from a pool of unidentified *Culex* (*Melanoconion*) mosquitoes, most likely *Cx*. *cedecei*, collected in Everglades National Park in 1964 [14]. It has more recently been isolated from a pool of *Cx*. *cedecei* from Manatee County, FL in 1993 [58]. SRV has also been found in Mexico and Guatemala [59]. It is not known to cause disease in humans.

Mahogany Hammock virus (MHV), is a subspecies of Guama virus (GMAV) and also a member of the genus *Orthobunyavirus* [51]. MHV was first isolated from the Everglades in 1964 from *Culex (Melanoconion) spp*. mosquitoes and cotton rat *Sigmodon hispidus* [13]. There have been no subsequent reports of this virus, and its public health potential is unknown. But, reports from Brazil indicate that two other Guama group viruses can infect humans and cause disease [60].

Gumbo Limbo virus (GLV), a subspecies of Marituba virus (MTBV), a member of the genus *Orthobunyavirus* [51]. It was first isolated in 1963 from *Cx*. *(Melaniconian) spp*. from Everglades National Park [15]. Other members of the Marituba species complex are found in South America (MTBV, Murucatu, Nepuya, and Restan) and can all cause human illness characterized by fever, headache, myalgia, and prostration [50].

St Louis encephalitis virus (SLEV) is a species in the Japanese encephalitis complex of the genus *Flavivirus* [61]. SLEV has caused epidemics in humans throughout the Americas [38,62]. Clinical manifestations include fever, headache, prostration, and central nervous system signs, including encephalitis and death [38]. Many infections are asymptomatic and more severe disease and fatalities generally occur in the older population. The finding of SLV in this study is not surprising as it is widespread within Florida which has experienced repeated human epidemics and recent seroconversions in sentinel chicken flocks in south Florida [63]. Our single isolate of SLEV came from *Culex nigripalpus*, the primary vector for this virus in Florida [64] and the most abundant mosquito in the Everglades (Fig 2). The isolate was from a hardwood hammock in Everglades National Park.

Other common arboviral infections of humans and wildlife in south Florida but not found in this study include West Nile virus (genus *Flavivirus*) and Eastern equine encephalitis (genus *Alphavirus*). Payhayokee virus (genus *Bunyaviridae)* was originally described from the Everglades in 1969 (14), but it was not found in this study.

Eastern equine encephalitis (EEEV) is the most virulent arbovirus affecting humans that occurs in Florida, with a case fatality rate of 30% [65]. It is widely distributed in the eastern US with constant year around transmission within South Florida, which is believed to be the source of seasonal transmission for northern latitudes [66,67]. *Culiseta melanura*, the enzootic vector of EEEV, was found at 7 collection sites during 2013, mostly (95%) in Fakahatchee. This species was not found at any site during 2014. *Culex erraticus*, the 5^th^ most abundant species we found (Fig 6), is also vector competent for EEEV transmission [68]. Human cases of EEEV infection have been reported from both Dade and Collier Counties, which include the Everglades.

The public health significance this diverse array of arboviruses in the Everglades is uncertain. In contrast to SLE, EEE, and WNV, relatively few cases of human disease caused by these lesser known arboviruses have been reported to date, despite their prevalence in hot spots known to be frequented by visitors and residents. Limited serological studies conducted in the past [10,39,52] have shown relatively high antibody rates to EVEV, KEYV, and TENV among humans, especially indigenous Amerindians living in the Everglades. These observations demonstrate the potential for human exposure to arbovirus infection in the Everglades. However, no recent serological studies in humans have been conducted.

Little is known of the importance of these arboviruses to the wildlife of the Everglades. Avian fatalities are known to be caused by EEE and WNV infection and antibodies to EVEV, TENV, SLE and KEYV have been reported from several species of birds and mammals [55,69–71]. Considering the prevalence of arboviruses reported here, studies on their effects upon wildlife of the Everglades are warranted.

### Potential drivers of arbovirus emergence

Our study provides baseline data on the distribution and abundance of mosquitoes and arboviruses among the dominant landscape features of the Everglades over two summer seasons with markedly different water levels. Changes in the current hydrological features of this region will impact arbovirus activity in ways that are poorly understood. However, change is inevitable and efforts should be made to understand and anticipate outcomes that could have significant impacts upon human and animal health in the region.

### Wetlands restoration

The most important environmental change currently affecting the Greater Everglades Region is the Comprehensive Everglades Restoration Program (CERP), a $10.5 billion water management program which is already in progress [72]. With a 35 year timeline, CERP is designed to restore the natural waterflow of the Everglades to pre-disturbance levels and also to manage water resources to prevent flooding and preserve the fresh-water aquifer. This massive water management project is anticipated to have major impacts upon the hydrology of the region with subsequent changes in vegetation and wildlife [7–9,73]. However, no consideration has been given to the impact of CERP upon mosquitoes or arboviruses, which could be profound. Hydrologic factors such as water depth, hydroperiod, etc. greatly influence aquatic larval habitat availability and affect the production of mosquitoes species differently [73,74]. The anticipated changes in the distribution and abundance of vertebrate wildlife species will also affect the availability of reservoir hosts for arboviruses [75,76]. The long-term effects of the CERP upon arbovirus activity in the Everglades are unknown, but the overall goal is to increase water flow into the southern Everglades region. Our data show that increased water levels which could result in conditions more favorable for both vectors and arboviruses.

Restoration efforts have been underway since 1991 in the Picayune State Forest, a former 29,000 ha. development where roads have been leveled and drainage canals are being filled [24,77]. The objective is to raise the water table 5 feet (1.5 m) to restore the surface sheet flow of 50 years ago. No arboviruses were found at 9 locations sampled there in 2014, but 6 isolations of 3 different arboviruses were obtained from the adjacent Fakahatchee Preserve during 2013. *Cx*. *cedecei* is currently rare in the Picayune but common in the Fakahatchee, even though the sampling locations are only 5 km distant. Continued restoration of the Picayune is anticipated to create water levels similar to the Fakahatchee, which will likely result in conditions more favorable for *Cx*. *cedecei* and the arboviruses associated with it.

#### Climate change

Climate change is also expected to influence the ecology of sub-tropical South Florida in ways that could also impact arbovirus activity [78,79]. Changes in rainfall patterns and sea level rise will augment the anticipated effects of CERP upon hydrology and will affect mosquito productivity. The anticipated increase in mean temperature will make the southern Everglades Region more tropical [80,81], providing an environment more favorable to invasive mosquito species from the tropics. Longer warm periods and shorter cold periods will also lengthen the transmission season for arboviruses by vector mosquito species [82–84]. Warmer temperatures generally decrease the extrinsic incubation period for arboviruses in mosquitoes, shortening the transmission cycle [85]. Changes in seasonal temperature can influence vertebrate host selection for some mosquito species, as has been observed with *Cx*. *erraticus*, that would also impact arbovirus transmission rates [84,86,87]. Alternatively, anticipated increased hurricane activity could temporarily reduce the abundance of some species, as has been observed with *Ae*. *taeniorhynchus* [88]. Climate change is considered to be a major driver of disease emergence in other ecosystems [82,89].

#### Invasive species

At least 8 new species of mosquitoes have become established in South Florida over the past 30 years [90–97]. All but one have neotropical origins and are presumed to have been introduced by natural means. Notably among these is *Culex panocossa* [87], a member of the *Melanoconion* subgenus. This recently established species is a competent vector of both VEEV and EEEV. It was discovered on the eastern boundary of Everglades National Park, close to where we isolated EVEV from *Cx*. *cedecei* and to where the first human case of EVEV infection was thought to have been acquired [49]. *Cx*. *panocossa* has an obligatory association with water lettuce (*Pistia stratiotes*) which is a dominant aquatic macrophyte inhabiting drainage and roadside canals in developed areas of South Florida. Because *Cx*. *cecedei* does not normally occur in developed areas, *Cx*. *panocossa* could serve as an important bridge vector of EVEV to humans. With the anticipation of a more tropical South Florida due to climate change, it is not unreasonable to expect future introductions of other exotic tropical mosquito species into South Florida, some of which may have vector potential for arboviruses.

Most of the arboviruses endemic to the Everglades are of tropical origin. With the exception of West Nile virus, no new zoonotic arboviruses have been found in the region since the original studies conducted in the 1960’s and 1970’s [11,13–15]. Novel arboviruses can be introduced by either an infected mosquito or an infected vertebrate host. Considering that South Florida is becoming increasingly tropical and a hub of international travel and commerce, future introductions of other tropical zoonotic arboviruses should be anticipated.

Over 150 exotic species of animals are considered to be invasive in south Florida [98] Such introductions can affect arbovirus transmission by altering the availability of mosquito blood meals from the endemic reservoir competent host species. For example, there is evidence that the introduced Burmese python (*Python bivittatus*), now abundant in the southern Everglades, may be increasing the prevalence of EVEV in *Cx*. *cedecei* by consuming and reducing the availability of medium-sized mammals, thereby increasing blood meal acquisition from reservoir-competent hosts of EVEV [18,99,100] Invasive vertebrate species may also directly change the blood-feeding patterns of mosquitoes when abundant [99,101].

#### Residential development

An estimated one million people move to Florida each year causing increased demand for residential development. While much of the Everglades Region is protected from such development, neighboring areas are becoming more susceptible to residential and commercial interests. As development approaches these natural areas, there will be increased human exposure to potentially infected mosquitoes. For example, a single residential development for 175,000 residents is planned immediately adjacent to the Fakahatchee Preserve where we obtained several isolates of Everglades, Shark River and Mahogany Hammock viruses [102]. Similar developments are planned for the eastern border of the Everglades National Park. It seems likely that such development will continue, resulting in many people living within the flight range of mosquitoes dispersing from these protected natural areas.

#### Limitations

We recognize several limitations to this study. There was uneven sampling of the different landscapes, which ranged from 37 samples (hardwood hammocks) to one (burned site), with a mean of 13.3 samples per landscape. Excluding the single burned site, the least sampled landscape (Fakahatchee, N = 5) still yielded 6 virus isolations, indicating that under-sampling was not a factor in detecting arbovirus activity among landscapes.

Also, different sites were sampled each year with a concentration in Everglades National Park and Fakahatchee in 2013 and Big Cypress National Preserve and Picayune in 2014. However, four sites with high arbovirus activity in 2013 and resampled in 2014 showed similar changes in mosquito species composition and low arbovirus activity with 11 isolates in 2013 and only one in 2014 The proportion of *Cx*. *cedecei* in the mosquito collections was also significantly reduced from 17% to <1% (S1 Fig).

The study was limited to just two years when water levels differed by only 20.2 cm (0.66 ft) in mid-summer (July). July maximum water levels have varied by as much as 76 cm (2.49 ft) deviation from a twenty year average [21]. This much variation could have a greater influence upon mosquito species composition and arbovirus activity than we observed.

## Conclusions

The Greater Everglades Region of subtropical South Florida supports a greater abundance and diversity of mosquito-borne arboviruses than other regions of North America. The arbovirus fauna of the Everglades is more like that found in tropical areas of South America [103,104]. Some of these endemic arboviruses are poorly known and their potential for causing disease in humans and wildlife is uncertain. This potential will likely be enhanced by the anticipated environmental changes that will alter the distribution and prevalence of arbovirus infection in mosquitoes. In our study, mosquito diversity and arbovirus prevalence were significantly higher when water level was also high (2013) and lower when water level was low (2014). Obviously, more than 2 years of observation will be required to firmly establish patterns in mosquito species abundance and the distribution of arboviruses. But based on our findings, it seems likely that anticipated future higher water levels will result in higher arbovirus activity. Additional studies are needed to further assess this potential and to monitor the impact of anticipated environmental changes in order to prevent future conflicts between public health and the protection of natural areas in the Greater Everglades Region.

## Supporting information

S1 Fig Mosquito species composition for six repeat collecting sites. (PDF)

S1 Table Collecting site locations. (XLSX)

S2 Table List of voucher mosquito specimens deposited. (XLS)

S3 Table List of virus isolations deposited. (XLSX)

S4 Table Mosquito collection data. (XLSX)

S5 Table Mosquito infection prevalence data. (DOCX)
