# Supplementary figures and images for: Emergence potential of mosquito-borne arboviruses from the Florida Everglades
Source: PLoS One. 2021 Nov 22;16(11):e0259419. doi: 10.1371/journal.pone.0259419 (PMC8608345; doi:10.1371/journal.pone.0259419)

Mosquito species composition of four repeat sites sampled during both 2013 and 2014

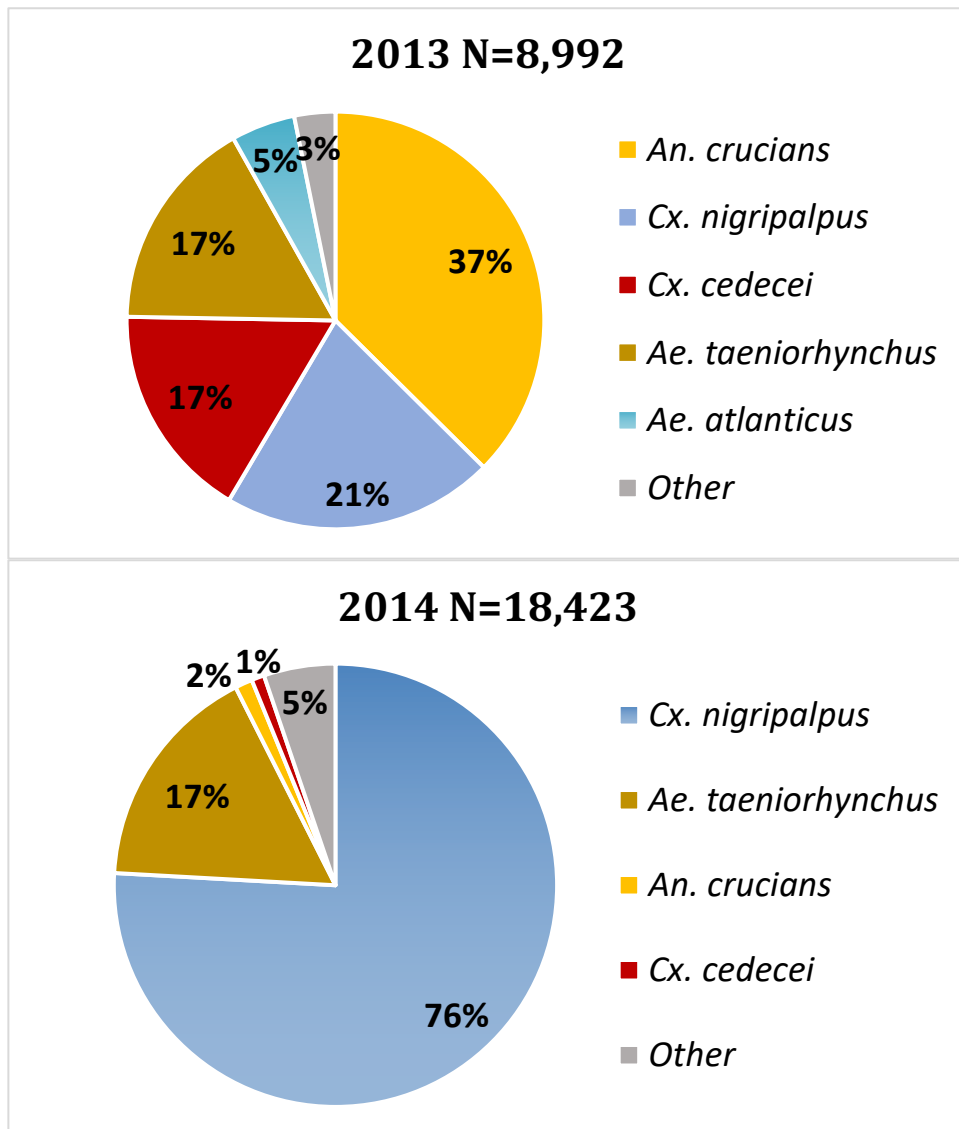

Supplement: S1 Fig — (PDF) [file pone.0259419.s001.pdf]
