# Supplementary material for: Emergence potential of mosquito-borne arboviruses from the Florida Everglades
Source: PLoS One. 2021 Nov 22;16(11):e0259419. doi: 10.1371/journal.pone.0259419 (PMC8608345; doi:10.1371/journal.pone.0259419)
Supplement: S5 Table — (DOCX) [file pone.0259419.s006.docx]

S5. Table. Infection prevalence of all arboviruses for each mosquito species.

| **Species** | **Rate** | **Lower Limit** | **Upper Limit** | **N Pools** | **N Pools+** | **N Individuals** |
| --- | --- | --- | --- | --- | --- | --- |
|  |  |  | **2013** |  |  |  |
| Ae atlanticus | 6.82 | 3.52 | 12.22 | 45 | 10 | 1662 |
| Cx atratus | 5.75 | 1.56 | 15.67 | 26 | 3 | 550 |
| Cx cedecei | 4.54 | 3.15 | 6.36 | 173 | 31 | 7508 |
| Wy mitchelli | 4.46 | 0.29 | 21.20 | 16 | 1 | 206 |
| Cx nigripalpus | 0.71 | 0.36 | 1.27 | 307 | 10 | 14272 |
| Cx erraticus | 0.70 | 0.04 | 3.42 | 46 | 1 | 1425 |
|  |  |  | **2014** |  |  |  |
| Cx cedecei | 13.51 | 3.51 | 38.75 | 25 | 3 | 262 |
| An atrops | 2.09 | 0.38 | 6.91 | 26 | 2 | 984 |
| An crucians | 1.15 | 0.47 | 2.39 | 128 | 6 | 5331 |
| Ae taeniorhynchus | 0.28 | 0.02 | 1.38 | 83 | 1 | 3513 |
